# Oculomotor-Related Measures Are Predictive of Reading Acquisition in First Grade Early Readers

**DOI:** 10.3390/vision9020048

**Published:** 2025-06-04

**Authors:** Avi Portnoy, Sharon Gilaie-Dotan

**Affiliations:** 1 School of Optometry and Vision Science, Faculty of Life Science, Bar Ilan University, Ramat Gan 5290002, Israel; avi.portnoy@biu.ac.il; 2 The Gonda Multidisciplinary Brain Research Center, Bar-Ilan University, Ramat Gan 5290002, Israel

**Keywords:** vision, oculomotor function, oculomotor dysfunction, reading, optometric evaluations, visual acuity

## Abstract

Some estimates suggest that one in seven good readers and the majority of children with reading difficulties suffer from oculomotor dysfunction (OMD), an umbrella term for abnormalities in comfortable and accurate fixations, pursuits, and saccades. However, national vision evaluation programs worldwide are often limited to distance visual acuity (dVA), not testing for OMD despite its high prevalence and the ease of detecting it in brief optometric evaluations. We hypothesized that reading acquisition is dependent on good oculomotor functions, and therefore inadequate oculomotor control will be associated with reading difficulties. We retrospectively examined and compared oculomotor evaluations (using DEM and NSUCO) and reading assessments (using standardized national reading norms) of a normative class (28 first graders (6–7 yr. olds)) that were independently obtained while blind to the other assessment. Better oculomotor performance as estimated by DEM was associated with better reading performance, and almost a third (29.6%) of the children were categorized by DEM as having OMD-related difficulties. Control analysis revealed dVA was not positively associated with reading performance. Linear regression analyses further corroborated these findings. Since this study is based on a small cohort and since there are studies suggesting that DEM may actually reflect visual processing speed or cognitive factors rather than oculomotor function, replications are needed to substantiate the direct contribution of oculomotor functions to reading acquisition. Young children struggling with reading may benefit from a comprehensive visual evaluation, including oculomotor testing, to provide a more thorough assessment of their learning-related difficulties.

## 1. Introduction

According to the Annie E Casey Foundation, in 2022, only 32% of children in fourth grade (9 year olds) in the US had reading skills that were proficient or above proficient level relative to their age [1]. Sociologist Donald Hernandez found that the risk of leaving school without a diploma (awarded for the successful completion of high school in the US) increases by 400% if a child enters their fourth year of formal education without proficient reading skills [2]. Acquiring reading skills would therefore seem key to young adults finishing high school.

While reading is often considered a high-level perceptual and cognitive task involving language and comprehension, reading also involves and relies on the efficient functioning of both the visual motor and visual perceptual systems. Reading demands high-level binocular control, ensuring that the two eyes remain converged on the plane of the printed page while simultaneously shifting the eyes from word to word, causing a relative asymmetrical adjustment in the angle of each eye. Similarly, the letters must be kept in focus, remaining sensitive to changes in accommodative demand as the eye shifts to lines closer or further from the reader. Accurate control of oculomotor functions such as fixation and saccades enables us to find and remain on the word currently being read, relative to the last word we have just read and the next word we are about to read. All these tremendously intricate and highly demanding oculomotor tasks are expected to take place with effortless automaticity simultaneously. These oculomotor functions take place concurrently with visual perceptual skills such as visual discrimination, visual memory, visual form perception, etc. While for most good readers this is typically achieved with precision, this is not always the case for poor readers.

Oculomotor dysfunction (OMD) is an umbrella term for abnormalities in comfortable and accurate control of the oculomotor system with respect to maintaining fixation and/or eye movements (saccades and/or pursuits) [3,4]. For those suffering from OMD, saccades can either overshoot (eyes land further than the designated target) or undershoot (eyes land short of the designated target). Both overshooting and undershooting are typically followed by smaller corrective saccades. In OMD, saccadic onset latency may sometimes be increased, and saccadic velocity may also be reduced. OMD may also be accompanied by unintentional motor responses to ocular movement (aka oculomotor overflow) of the jaw, tongue, head, or body when planning or performing pursuits or saccadic movements. The extreme demands on the visual system that reading entails may incapacitate a new reader, with potential impact on both reading speed and accuracy. OMD’s prevalence in school-aged normative readers is estimated at 22–24% [5,6], compared to estimations as high as 96% in child populations diagnosed with a reading disability [5,7]. It has been suggested that OMD may be linked to a lack of effective integration of sensory input, higher-level decision-making, and appropriate oculomotor response [3,8,9].

There are studies supporting a connection between ocular control and reading ability (e.g., [10,11,12,13,14,15] but see [16]), however, in many European countries as well as in Israel and the USA, visual evaluations at early ages do not standardly assess oculomotor control and include only distance visual acuity (dVA) [17]. In addition, many of the studies evaluating the relationship between reading and oculomotor control focus on children that read left-to-right languages and often categorize the children by their reading ability (e.g., those with difficulties and those that are more normative) and then test oculomotor function. As we hypothesized that oculomotor skills, and particularly fixations and saccades, are critical for acquiring comfortable and efficient reading skills, we retrospectively examined oculomotor performance in a normative class of first graders (6–7 year olds, *n* = 28) and tested whether these oculomotor skills can predict independently obtained reading evaluations (Hebrew, read right-to-left). We assumed that examining a normative class of first graders will allow us to obtain some approximation, even if coarse, as to the prevalence of oculomotor difficulties at that age. For control, we also compared visual acuity assessment with the reading evaluations. The optometric evaluation was performed by an optometrist blind to the reading evaluation results; reading was evaluated by an experienced reading teacher blind to the optometrist’s evaluations.

## 2. Methods and Materials

### 2.1. Participants

This study is retrospective, based on data obtained from twenty-eight first-grade children (6–7 year olds, 11 girls and 17 boys) that were all studying in the same class in a school that is part of the public education system in Israel.

The data from the optometric evaluations were obtained as part of the community outreach optometric evaluation activities routinely performed free of charge by the Bar-Ilan University School of Optometry and Vision Science (e.g., in schools, orphanages, centers for the elderly, immigration centers, and additional community centers in rural and peripheral parts of Israel, as well as in Bar-Ilan University Optometry clinics). The study protocol was in accordance with the guidelines of the institutional Bar-Ilan University ethics committee that approved retrospective analyses of data (stripped of identifying information) collected in optometric evaluations as well as scholastic evaluations to investigate the relationship between visual functions and scholastic performance.

The parents of the children, all in the same class, were offered the opportunity for their children to undergo an optometric evaluation in school and were asked to provide their consent in writing. Out of a class of thirty children, all but the parents of two children provided written informed consent that their child participate in the optometric evaluation (this was the initial cohort of 28 children from which an additional child was excluded from the study; see details below). The visual function evaluation was performed by a certified optometrist and took roughly twenty to thirty minutes per child. Children who already had a prior refractive correction wore their current daily glasses for each of the optometric tests. Parents were notified about any suspicious finding regarding their child’s vision that may require further evaluations. If visual acuity was found to be 6/12 or worse in either eye with their current spectacle correction (*n* = 4), after the visual assessment the child was referred to an optometrist for further refractive testing or an ophthalmologist for a full ocular health exam (based on retinoscopy results).

In addition, as part of the school program, all the children underwent a comprehensive reading examination, including reading speed and accuracy, performed by a reading teacher a couple of weeks after the optometric evaluation. Importantly, the teacher was not informed of the optometric results until a few weeks after the whole class’s reading assessments were completed, and the optometrist was not informed of the reading assessment results until after the optometric evaluations were completed. One child was excluded from the analysis due to significant language and learning difficulties observed independently in the optometric and reading evaluations. Another child’s dVA records were missing, and we therefore had to remove him from the dVA related analyses. Thus, the final cohort for the study was of 27 children (see descriptive statistics in Table 1 and individual data at https://osf.io/shw2v/, accessed on 30 May 2025) with one of these children missing dVA data.

### 2.2. Visual Evaluation

The visual function evaluation was performed as part of the community outreach optometric evaluation activities the Bar-Ilan University School of Optometry and Vision Science engages in (see details above). Each child’s optometric evaluation included the following standardized optometric tests carried out in this order (more details below): Distance and Near visual acuity (VA) [18,19], objective refraction—retinoscopy [18], cover testing (distance and near) [18], negative fusional vergence (NFV) at near (prism bar) [18], positive fusional vergence (PFV) at near (prism bar) [18], near point of convergence (NPC) [18], stereopsis [20], accommodative amplitude [21] (right eye (RE) only [22]), accommodative facility [18] (RE only [23]), NSUCO [4], King Devick [24,25] and Developmental Eye Movement test (DEM) [15,26,27,28]. Lighting was provided by overhead fluorescent fixtures as well as sunlight from windows. For the dVA testing (because a projector was used), the fluorescent lighting was extinguished, and the sunlight was partially blocked by curtains, leaving the room moderately lit [29].

Distance visual acuity (VA) was performed using a standard Snellen acuity chart using numerical optotypes projected onto a wall 6 m from the child and performed both monocularly and binocularly [18]. Since reading is performed with both eyes, the binocular distance VA measurement was used in the analyses of this study.

Near VA was assessed at 40 cm monocularly and binocularly using a standard near VA test card with numerical optotypes [18] (Rosenbaum pocket vision screener).

Retinoscopy is an objective assessment of the child’s refractive status. This was measured to ensure that children not wearing glasses were not in need of any refractive correction and that children wearing glasses were not under- or overcorrected by more than 0.5 diopters (D) of myopia (short-sightedness), 1.5 D of hyperopia (far-sightedness), or 0.75 D of astigmatism [23]. If a discrepancy equal to or above these refractive errors was found, they were referred to get corrective spectacles (*n* = 4).

The Developmental Eye Movement (DEM) test was one of the tests used to assess oculomotor function and has vertical and horizontal subtests. The DEM is composed of digits organized in vertical or horizontal alignments (each arrangement in a different subtest, vertical assumed to predominantly rely on vertical saccades, horizontal assumed to rely predominantly on horizontal saccades) that the examinee has to read aloud, and the scores are given according to speed and accuracy performance in each of these subtests. The time recorded for each subtest is adjusted based on the number of digits that were skipped or added by the examinee. An overall categorization of performance is then defined using the ratio of horizontal test speed divided by vertical test speed (assumed to be related to language-related processes), where the purpose of the ratio score is to differentiate language automaticity difficulties (rapid automatized naming (RAN)) [30] from oculomotor difficulties [27], as explained in the DEM manual [31]. Results are categorized into one of the four following types [27,31]:

1. Normal language automaticity and oculomotor function.
2. Normal language automaticity with oculomotor dysfunction.
3. Abnormal language automaticity function but normal oculomotor function.
4. A combination of both oculomotor and language automaticity dysfunctions.

DEM speed and accuracy raw scores are standardized according to age or grade (grade used in this study, see individual data at https://osf.io/shw2v/, accessed on 30 May 2025) with a mean score of 100 and standard deviation of 15 (higher values correspond to better performance).

The NSUCO (Northeastern State University College of Optometry)/Maples oculomotor test was carried out according to the manual supplied by the Optometric Extension Program (OEP) [9]. This test is nonverbal, testing both saccades and pursuits individually. The examinee is asked to follow targets that are held at different specific locations or moved according to specific spatial trajectories by the optometrist. Targets are round, reflective, and approximately 0.5 cm in diameter. Targets are positioned between 40 cm and the Harmon distance (distance from elbow to middle knuckle) of the examinee. The distance between targets or diameter of pursuits should be 10 cm in each direction from the examinee’s midline (nose). This test grades eye movement (saccade and pursuit) ability, accuracy, and head movement and body movement accompanying the eye movements. Each of the sub-scores is graded from 1 (poor performance) to 5 (good performance). Two sub-scores titled ‘head movement’ and ‘body movement’ are graded by how much head or body movement is triggered by ocular movement. This indicates an inability to dissociate ocular movement from head or body movement, which is a typical sign of OMD [9]. A grade of 1 represents gross head or body movement (poor performance), and a grade of 5 represents no head or body movement (good performance). More detailed explanations of the scoring can be found elsewhere [9].

### 2.3. Reading Assessment

Reading of each child was tested by an experienced reading teacher. The teacher used the age-based norm-referenced tests in Hebrew provided by the Israeli Ministry of Education [32] (see more details at https://web.archive.org/web/20221127093308/https://cms.education.gov.il/EducationCMS/Units/Rama/AarachaBeitSifrit/Mivdak_Hebrew.htm, accessed on 27 November 2022) and followed their fixed protocol for testing. The assessment [33] comprised 10 different subtests assessing reading and writing skills, including accuracy, speed, phonological awareness, and reading comprehension.

In our retrospective analysis, we chose to focus on subtest 8, which assesses reading speed and accuracy (but not comprehension), since it best simulates visual demands of daily classroom reading. Subtest 8 is based on a unique text (not related to any of the other subtests) of a 77-word story presented in 8 widely spaced lines. The child is required to read it aloud while the teacher evaluates both accuracy and speed. The teacher marks all the words incorrectly read by the child, including self-corrections. According to the national standards [32], a score of 69–77 words read correctly is categorized as a ‘pass’, a score of 58–68 words read correctly as ‘demands follow-up’, and a score of or below 57 words read correctly as a ‘fail’. With regards to reading speed, the teacher measures the amount of time it takes the child to read the 77 words. A score of 195 s or less to read the text is considered a ‘pass’, a score of 196 to 265 s is considered ‘demands follow-up’, and a score of 266 s or slower is deemed a ‘fail’ [32]. For control, we also examined children’s performance on subtest 7 (reading unfamiliar words), and more information on that can be found at https://osf.io/shw2v/, accessed on 30 May 2025).

### 2.4. Analyses

For each child, the measured DEM speed (duration in sec) and the number of errors for each of the tests (A, B, C—two vertical and one horizontal) were inserted into the DEM Scorer Optometric Clinical Software Version 2.2 (© 2008 Software in Motion) that converted each measure to standardized scores (according to the child’s grade level following the guidelines provided in the DEM manual [31]). The corresponding standard scores, DEM type categorization, and the DEM ratio were calculated by the program (data available at https://osf.io/shw2v, accessed on 30 May 2025). Spearman rank order correlation analyses between reading performance (speed, accuracy) and visual functions (DEM standardized speed or accuracy and dVA) were run to evaluate whether there was a monotonic relationship between reading and a specific visual function. Comparisons of reading scores of the poorer visual performers and those of the better visual performers were also performed: for each visual function, the class was divided into two groups based on their visual performance (a group of higher visual scores and a group of lower visual scores, see more details below per visual function). The reading scores of the two groups were then subjected to a non-paired, one-tailed *t*-test (unequal variance; the *t*-test function in R was used, and this function relies on the Welch (or Satterthwaite) approximation for the degrees of freedom for *t*-tests with unequal variance (more details on the R statistical package we used appear below)). We also present the distribution of the reading test outcomes (pass, demands follow-up, or fail) for the better and poorer visual function groups. For the DEM, we also added ROC-based analysis (see below). Lastly, we ran a set of linear regression analyses to assess whether reading performance was predicted by DEM speed, accuracy, type, and/or dVA. For one child, data for dVA was missing, and we therefore removed this child’s data for all the linear regression models so we could also have a multifactorial model.

Dividing the children according to higher and lower visual score groups. For the DEM, we split the children according to (i) faster vs. slower horizontal speed performance (standardized scores), (ii) faster vs. slower vertical speed performance (standardized scores), (iii) more vs. less accurate performance (standardized scores based on errors), and (iv) normative vs. oculomotor-related difficulties as determined by DEM Type (i.e., Type 1 (normal) vs. Types 2 and 4 (oculomotor-related difficulties)). Because our cohort size contained an odd number of children (*n* = 27), for DEM analyses (i)–(iii), after sorting the class according to that DEM score, the 14th child was removed prior to analyzing the data. This enabled us to have two groups of equal size. In addition, since halving the class was not based on normative performance criteria, for the DEM (i)–(iii) analyses we also applied the −1 SD cut-off (i.e., splitting the class according to those below −1 SD from the mean of the standardized scores (likely to have a difficulty) and those not below −1 SD (likely without difficulty)). For these DEM analyses ((i)–(iii) with the −1 SD cut-off), since they were rather continuous, we also performed an ROC-based analysis to assess whether the DEM test effectively differentiates children with and without reading difficulties. Difficulty in reading (“positive”) was defined for “demands follow-up” or “fail” scores (see above for more details), and the DEM standardized score varied to obtain for each threshold true positive and false positive rates, from which the ROC curve was created (see more details in https://osf.io/shw2v/, accessed on 30 May 2025). For the NSUCO, the children were split by their pass or fail scores according to age-based norms. For VA, analysis was based on the group of children with normal VA (“6/6”) vs. the group of children with below-normal VA.

All of the above analyses were performed with R Studio (RStudio 2022.02.3 Build 492, © 2009–2022 RStudio, PBC) running with R version 4.2.1 (released on 2 June 2022, aka Funny-Looking Kid, Copyright © 2022 The R Foundation for Statistical Computing, Platform: ×86_64-w64-mingw32/×64 (64-bit)). See https://osf.io/shw2v/ (accessed on 30 May 2025) for more detailed data and additional supplementary information.

## 3. Results

Based on years of clinical observations, we hypothesized that reading acquisition would be highly dependent on oculomotor functions and therefore examined the relationship between OMD-related measures and reading acquisition scores in a normative group of first graders.

### 3.1. Oculomotor Evaluation by DEM and the Relation to Reading Performance

The developmental eye movement test (DEM) is a well-established quick optometric evaluation tool whose last section (test C with horizontal arrangement of numbers) is considered to mimic the saccadic demands of reading on the visual system [15,26,27,34,35,36]. We therefore expected that DEM performance on horizontal test C would be predictive of reading performance during the reading acquisition period in either speed, accuracy, or both.

**Figure 1:**
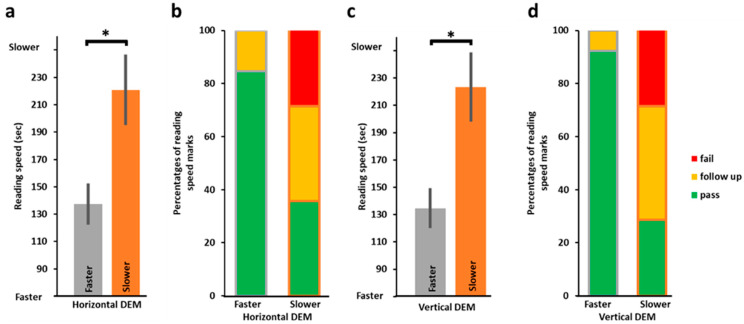
Faster reading speed and better reading grades for faster DEM performers (*n* = 26). (**a**) Average reading time in seconds for slower (orange, on right, *n* = 13) and faster (grey, on left, *n* = 13) horizontal DEM performers; the faster horizontal DEM performers were found to be significantly faster in their reading performance (*t*(18.54) = 2.63, *p* = 0.008, 1-tailed). DEM speed performance is standardized and based on the duration in seconds to complete the task (see https://osf.io/shw2v/ (accessed on 30 May 2025) for the individual data and corresponding standardized scores). (**b**) Proportions of reading speed categorization (pass in green, demands follow-up in yellow, or fail in red) for the slower DEM performers (*n* = 13, right) and the faster DEM performers (*n* = 13, left). (**c**,**d**) Same analysis as in (**a**,**b**) but for vertical DEM speed, the faster vertical DEM performers were found to be significantly faster in their reading performance (*t*(18.53) = 2.76, *p* = *0*.006, 1-tailed). Note that there were no fails for the reading speed test in both the faster vertical and faster horizontal DEM groups. Asterisks denote *p* < 0.01, error bars, SEM.

We started by examining the relationship between DEM horizontal speed and reading speed. To that end, we first correlated (Spearman rank correlation) DEM speed (standardized scores) with reading speed and found DEM speed to be significantly predictive of reading speed (horizontal *r_s_*(25) = −0.43, *p* = *0*.012), as we hypothesized (analyses with DEM raw speed scores were almost identical). In addition, we also compared the reading speed scores of slower DEM performers to those of faster DEM performers and found (Figure 1a) that the faster DEM group (as determined by the horizontal test C speed (standardized scores), in grey in Figure 1a) was significantly faster on the reading test than the slower DEM performers (in orange, *t*(18.54) = 2.63, *p* = 0.008, 1-tailed). Additional analysis splitting the group using a cut-off threshold of −1 SD for the standardized DEM horizontal speed scores (one group consisting of children whose scores were below −1 SD from the mean and the second group all children who performed above this threshold) replicated these findings with the above −1 SD group performing significantly better on the reading speed than the below −1 SD group (reading speed of the below −1 SD DEM horizontal group (*n* = 13): 220.79 s ± 25.80 (SEM), of the above −1 SD DEM horizontal group (*n* = 14): 137.38 s ± 15.17 (SEM), *t*(20.85) = −2.79, *p* = 0.006). In addition, as can be seen in Figure 1b, in the faster DEM group (test C), the proportion of ‘pass’ grades in reading speed was high (>80%) with no ‘fail’, while in the slower DEM group, only about a third (38.5%) received ‘pass’ in reading speed and 30.8% ‘fail’. The results of the vertical DEM test were similar and are presented in Figure 1c,d (correlation of DEM vertical speed and reading speed: *r_s_*(25) = −0.51, *p* = 0.003; reading speed of faster vs. slower DEM vertical groups: *t*(18.53) = 2.76, *p* = *0*.006, 1-tailed; faster DEM group: 92% ‘pass’ in reading speed and 0% ‘fail’, slower DEM group: 30.8% passed, 23.1% failed). When partitioning according to −1 SD cut-off threshold of the DEM vertical speed, the results were marginally replicated with the reading speed of the below −1 SD DEM vertical group (*n* = 11, 216.9 s ± 32.22 (SEM)) marginally slower than that of the above −1 SD group (*n* = 16, 155.69 s ± 16.49 (SEM); *t*(15.23) = −1.69, *p* = 0.056).

**Figure 2:**
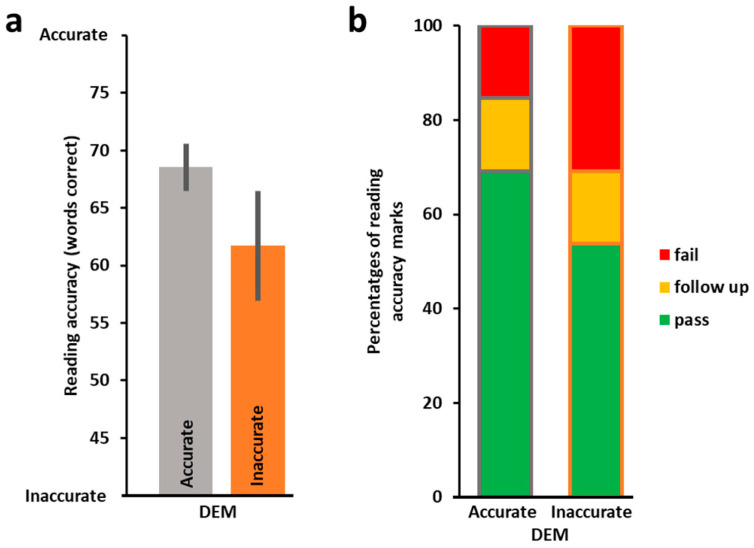
Reading accuracy according to DEM accuracy (standardized, based on error scores) (*n* = 26). (**a**) Average number of words read correctly (out of 77) by the more accurate (grey, *n* = 13, left) vs. the less accurate (orange, *n* = 13, right) DEM performers were not significantly different (*t*(16.264) = −1.31, *p* = 0.104, 1-tailed). DEM accuracy performance is standardized and based on the number of errors made (see https://osf.io/shw2v/, accessed on 30 May 2025, for the individual data of errors and corresponding standardized accuracy scores). (**b**) Proportions of reading accuracy categorization scores (pass in green, demands follow-up in yellow, fail in red) by DEM accuracy performance. These data tend to suggest that the more accurate DEM group (left column) may receive better reading grades. Error bars, SEM.

Next, we examined the relationship between DEM accuracy (number of errors made on the DEM) and reading accuracy. DEM horizontal accuracy and reading accuracy were not correlated (*r_s_*(25) = 0.18, *p* = 0.179). The more accurate DEM group was on average more accurate on their reading, but this did not reach significance (*t*(16.26) = −1.31, *p* = 0.104, 1-tailed, see Figure 2a). This was reflected in better reading grades (see Figure 2b, the more accurate DEM group with 69.2% passes in reading accuracy and only 15.4% fails, and the less accurate DEM group with 53.8% passes and 30.8% fails). Additional analysis was performed splitting the group using a cut-off threshold of −1 SD for the standardized DEM accuracy scores (one group consisting of children whose scores were below −1 SD from the mean and the second group consisting of children who performed above this cut-off threshold) replicated these findings (mean reading accuracy of the below −1 SD DEM accuracy group (*n* = 7): 58.14 words ± 8.45 (SEM); of the above −1 SD DEM accuracy group (*n* = 20): 67.3 words ± 1.73 (SEM), *t*(6.51) = 1.062, *p* = 0.16).

**Figure 3:**
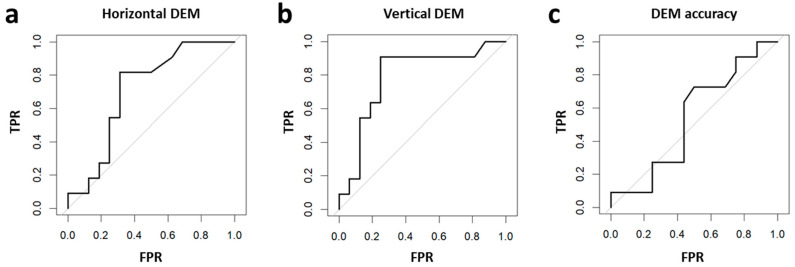
Difficulty in reading (−1 SD) based on DEM performance: ROC analysis. True positive rate (TPR, y-axis) as a function of false positive rate (FPR, x-axis) for predicting difficulty in reading based on DEM performance. Difficulty in reading was defined for “demands follow-up” or “fail” reading scores, which correspond to −1 SD or worse performance. (**a**) DEM horizontal speed performance significantly differentiated children with vs. children without reading speed difficulty (AUC = 0.7074, 95% CI: 0.5049–0.9098). (**b**) DEM vertical speed performance significantly differentiated children with vs. children without reading speed difficulty (AUC = 0.7869, 95% CI: 0.5959–0.9779). (**c**) DEM accuracy did not successfully differentiate children with vs. children without reading accuracy difficulty (AUC = 0.5398, 95% CI: 0.3113–0.7682). See Results for further details.

ROC curve analyses were also applied to assess whether the DEM test speed and accuracy measures can effectively differentiate children with and without reading difficulties (see Methods for more details). As can be seen in Figure 3, the results of these analyses show that DEM speed measures were effective in differentiating children with and without reading difficulties, but DEM accuracy was not. Specifically, DEM horizontal speed discriminative ability (for differentiating children with and without reading speed difficulty) was statistically significant, with area under the curve (AUC) significantly above chance (AUC = 0.7074, 95% CI: 0.5049–0.9098 (DeLong)). DEM vertical speed discriminative ability was also significantly above chance (AUC = 0.7869, 95% CI: 0.5959–0.9779 (DeLong)), but DEM accuracy discriminative ability (for differentiating children with and without reading accuracy difficulty) was not significant (AUC = 0.5398, 95% CI: 0.3113–0.7682 (DeLong)).

**Figure 4:**
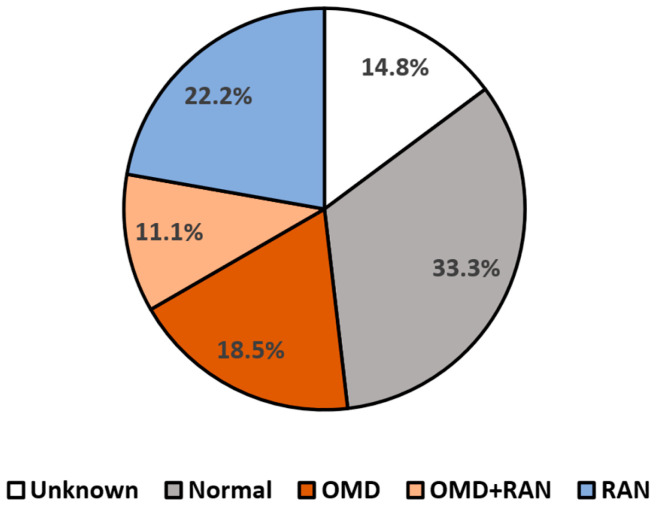
Percentages of children found in each DEM classification type (*n* = 27). The grey section (33.3%, *n* = 9) represents normal language automaticity and oculomotor function (DEM Type 1). Orange shades represent DEM types associated with OMD (29.6%, *n* = 8), with darker orange (18.5%, *n* = 5) representing children categorized by DEM as having normal language automaticity with oculomotor dysfunction (Type 2) and lighter orange section (11.1%, *n* = 3) representing DEM performance categorized as a combination of both oculomotor and language automaticity dysfunctions (Type 4). The blue section (22.2%, *n* = 6) represents abnormal language automaticity function but normal oculomotor function (Type 3). The white section (14.8%, *n* = 4) represents DEM performance that cannot be categorized according to the DEM protocols, possibly due to other visual factors such as accommodative or binocular dysfunctions.

**Figure 5:**
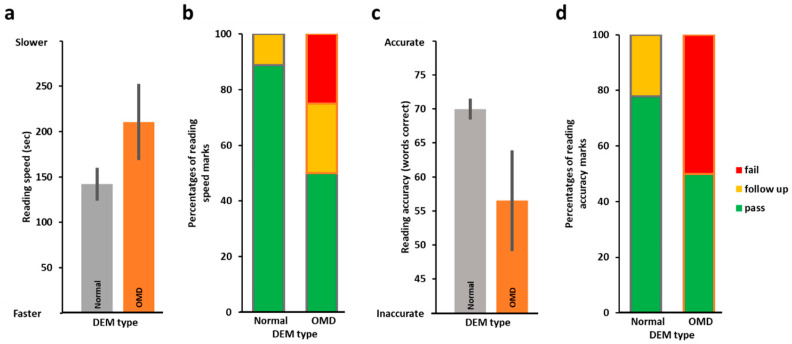
Reading speed and accuracy performance according to DEM Type categorization (*n* = 17). (**a**) Average reading speed in seconds as performed by the normal DEM group (grey Type 1 on left, *n* = 9) and the OMD DEM groups (orange, Types 2 and 4 on right, *n* = 8). As can be seen, the normal DEM group was on average faster in their reading performance (marginally significant, see Results). (**b**) Proportions of reading speed categorization (‘pass’ in green, ‘demands follow-up’ in yellow, or ‘fail’ in red) for the normal DEM group on left and the OMD DEM groups on the right. (**c**,**d**) Same analysis as in (**a**,**b**) but for reading accuracy. Note that there was no fail for either reading speed or accuracy in the normal (Type 1) DEM group. Error bars, SEM.

To investigate the relationship between the overall DEM categorization (related to the ratio, see Methods) and reading performance, we first examined the distribution of the DEM classification types amongst all the children in the study (see Figure 4) and found that ~29% were categorized by the DEM as having OMD (DEM Types 2 and 4), while a third were categorized as having normal oculomotor function (Type 1). We then tested whether children categorized as having OMD (Types 2 and 4, *n* = 8) had worse reading performance than the children categorized by the DEM as having normal oculomotor function (Type 1, *n* = 9). Reading speed was on average slower in the children categorized as having OMD (DEM Type 2 and 4 groups, *n* = 8) relative to the children categorized as having normal oculomotor function (DEM Type 1 group, *n* = 9), but this difference was only marginally significant (*t*(9.54) = −1.5, *p* = 0.083, 1-tailed, see Figure 5a). Reading accuracy was also worse in the children categorized as having OMD (DEM Type 2 and 4 groups, *n* = 8) relative to the children categorized as having normal oculomotor function (DEM Type 1 group, *n* = 9), but this difference was only marginally significant (*t*(7.61) = 1.78, *p* = 0.057, 1-tailed; see Figure 5c). In the normal oculomotor group, for both reading speed and reading accuracy, the proportions of ‘pass’ grades were high (~80%), and there were no ‘fail’ grades (presented in red in Figure 5b,d). In contrast, for the OMD groups, between a quarter and 50% failed (see Figure 5b,d). Six participants were categorized as Type 3 (language automaticity difficulties), and four participants did not fit into any of the four DEM categorization types and were categorized as an unknown type.

In summary, the DEM speed results for both vertical and horizontal tests were significantly predictive of reading speed. DEM type was marginally predictive of both reading accuracy and reading speed. DEM higher accuracy was also on average associated with higher reading accuracy, but this was not statistically significant. Interestingly, DEM horizontal speed was correlated with DEM accuracy (standardized scores, *r* = 0.5528, *t*(23) = 3.128, *p* < 0.005, two outliers excluded), and reading speed scores were correlated with reading accuracy (*r* = −0.6242, *t*(24) = −3.915, *p* < 0.001, one outlier excluded), indicating that children who were faster were also more accurate on either test (more details in https://osf.io/shw2v/, accessed on 30 May 2025). In addition, as control, we also tested whether DEM speed was predictive of reading speed for unfamiliar words (subtest 7), and these results (which did not come out as significant) can be found at https://osf.io/shw2v/ (accessed on 30 May 2025).

### 3.2. Oculomotor Evaluation by NSUCO and the Relation to Reading Performance

**Figure 6:**
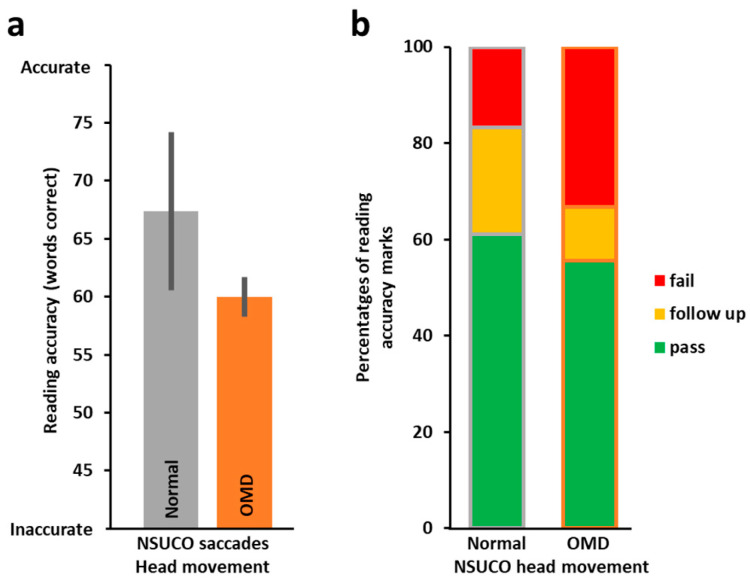
Reading accuracy performance according to standardized optometric NSUCO subscore that grades the amount of head movement resulting from saccades (*n* = 27). (**a**) Average number of words read correctly as performed by the age-appropriate NSUCO head movement group (grey, *n* = 15) and below age-appropriate (orange) group (orange, *n* = 12). The age-appropriate group was more accurate on average in their reading. (**b**) Proportions of reading accuracy categorization (‘pass’, ‘demands follow-up’, or ‘fail’) for both groups. Error bars, SEM.

In addition to the assessment of oculomotor skills by the DEM, oculomotor skills were also assessed independently by the well-established optometric NSUCO oculomotor test. The NSUCO test assesses the accuracy of both pursuits and saccades, and we hypothesized that since reading predominantly involves static stimuli and saccades, these would be related to reading accuracy. NSCUO scores four aspects of saccadic performance: ability (typically at ceiling performance), accuracy (ability to refrain from overshooting or undershooting when trying to reach targets), head movements, and body movements. As body movements accompanying saccades usually involve head movements as well (but the opposite is not always the case), we examined saccadic head movement scores and their relationship to reading ability. When we split the class according to the pass/fail criterion for the saccadic head movement sub-score, we found the NSUCO performance was not significantly predictive of reading accuracy (*t*(9.039) = −1.05, *p* = 0.160, 1-tailed). As can be seen in Figure 6a, the group that did not succeed on the NSUCO saccadic head movement performance (*n* = 12, in orange on right) performed on average less accurately on the reading accuracy test than the group that achieved age-appropriate scores on the NSUCO saccadic head movement performance (*n* = 15, in grey on left). We then compared proportions of ‘pass’, ‘demands follow-up’, and ‘fail’ grades in the reading accuracy test for the NSUCO age-appropriate head movement group (*n* = 15, Figure 6b on left) and for the below age-appropriate group (*n* = 12, Figure 6b on right). The proportion of reading accuracy ‘pass’ grades in the NSUCO age-appropriate head movement group was 61.1% and 16.7% with a ‘fail’. The below age-appropriate NSUCO group had a 56% ‘pass’ rate for reading accuracy and a 33.3% ‘fail’ rate (Figure 6b).

### 3.3. Distance Visual Acuity (VA) and the Relation to Reading Performance

**Figure 7:**
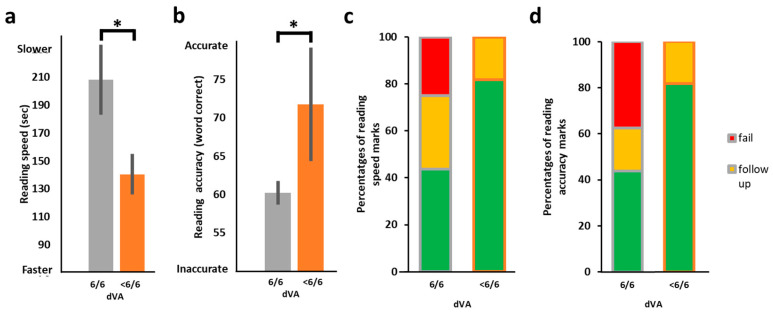
Reading performance in children with better and poorer dVA (*n* = 26). (**a**) The Y-axis represents reading time in seconds. The right orange bar depicts reading speed (in words per minute) of the below-average dVA (<6/6) group (*n* = 10), and the left grey column that of the average dVA (6/6) group (*n* = 16). Reading speed of the better acuity group was significantly *slower* than that of the poorer acuity group (*t*(23.284) = −2.22, *p* = 0.037, 2-tailed). (**b**) Similar to (**a**) with Y-axis representing the number of words read accurately out of the 77 words in the test. Reading accuracy performance of the better acuity group was significantly *less* accurate than that of the group with less than average (<6/6) dVA (*t*(17.363) = 2.82, *p* = 0.012, 2-tailed). (**c**) The stacked bar chart displays proportions of reading speed categorization (‘pass’ in green, ‘demands follow up’ in yellow, ‘fail’ in red), right bar—reading score distribution of the less than average dVA group, left bar—similarly for the normal dVA group. Note that there were no ‘fails’ in the <6/6 group and the ‘pass’ rate was higher than in the 6/6 group. (**d**) Same analysis as in (**c**) but for reading accuracy. Asterisks, *p* < 0.05, error bars, SEM.

As control, we also assessed whether non-oculomotor visual measures may be associated with reading acquisition performance. We compared distance VA, a commonly used measure to assess vision in school-aged children, with reading performance (speed and accuracy). Surprisingly, when we correlated dVA with reading speed, we found that poorer dVA was marginally predictive of faster reading (*r_s_*(24) = 0.37, *p* = 0.062) as well as being significantly predictive of more accurate reading (*r_s_*(24) = −0.47, *p* = 0.014). When comparing the reading of the better dVA group with the poorer dVA group, we found that children with worse dVA (less than 6/6, *n* = 10) were significantly faster in their reading performance (*t*(23.284) = −2.22, *p* = 0.037, 2-tailed; Figure 7a) and significantly more accurate in their reading (*t*(17.363) = 2.82, *p* = 0.012, 2-tailed) than children with normal dVA (6/6, *n* = 16, see Figure 7b). Amongst children with normal dVA (i.e., 6/6), the proportions of ‘pass’ for the reading speed and accuracy were lower than in the group with reduced dVA (i.e., less than 6/6). There were no ‘fail’ in the reduced dVA group, while in the normal dVA group there were >30% ‘fail’ marks (Figure 7c,d). It is important to note that many of the participants with reduced dVA (less than 6/6) had only marginally reduced dVA. Six children with poorer dVA also had uncorrected myopia and/or astigmatism. Our results indicate that dVA, which we used as a control, was not a good predictor of reading performance from a close distance.

### 3.4. Linear Regression Analyses

To further corroborate our findings, we also ran linear regression analyses assessing whether aspects of the DEM and/or dVA were predictive of reading accuracy or speed. As can be seen in Table 2 (top part, reading accuracy), dVA (Model 1) on its own was not significantly predictive of reading accuracy, but DEM horizontal accuracy was (see Model 2), and so was the DEM ratio (Model 3). We also tested whether all predictors combined (dVA, DEM accuracy, and DEM ratio) may better explain reading accuracy (Model 4), but this model did not outperform Model 3 based on the DEM ratio alone (compare Model 3 with *R^2^* of 0.2394 vs. Model 4 with *R*^2^ of 0.2122). Reading speed analyses (Table 2, lower part) revealed that dVA alone (Model 1) was also not significantly predictive of reading speed, but DEM horizontal speed (Model 2) or DEM vertical speed (Model 3) on their own were significantly predictive. Importantly, the model that outperformed all models in explaining reading speed was Model 4, which combined dVA, DEM horizontal and DEM vertical speed, which were all significant with *R*^2^ of 0.4353. This indicates that the combination of both dVA and DEM speed measures gave the best prediction for reading speed.

## 4. Discussion

In this study, we retrospectively investigated whether oculomotor performance as estimated by two different clinical tests in a normative class of Hebrew (read right to left) speaking first graders is associated with reading acquisition performance. We found that both the vertical and the horizontal speed scores of a well-established test for oculomotor skills, the DEM, were significantly predictive of Hebrew reading performance. The DEM also suggested the potential presence of OMD-related difficulties in ~29% of the children. For an additional test routinely used in the optometry clinic, the NSUCO, the head movement sub-score of the test was on average indicative of early Hebrew reading acquisition ability, but this was not significant. Interestingly, better distance visual acuity was not positively associated with better reading performance.

Many studies have assessed reading abilities in different developmental conditions (ASD [37], attention-related disorders [38], developmental dyslexia [15,39], and auditory processing disorder [40]). Here we investigated the relationship between reading acquisition and developmental visual skills, focusing on the general population (normative class within the state educational system) rather than on specific developmental subgroups. We found a clear relationship between oculomotor-related measures (as estimated by the DEM) and reading acquisition, and the prevalence of OMD (as estimated by the DEM) was ~29%, which is in line with earlier estimates of 15–24% [5,6] prevalence in good readers and much higher prevalence in poor readers [5,7]. While our investigation was of a normative class in the general population, we cannot rule out the possibility that some of the children may have had reading-related or attention-related difficulties alone or in addition to visual issues, given the prevalence of these conditions in the general population (5–10% [41] and roughly 5% [42], respectively).

It is unclear if the proportion of OMD (as estimated by the DEM) in the class we examined truly reflects OMD prevalence in the entire population. DEM is a clinical tool that relies on verbal reports to estimate visual perception (of the digits being scanned) but does not directly measure eye movements, and therefore its categorization of OMD (i.e., DEM Types 2 and 4) should be viewed as suggestive rather than definitive. Furthermore, the overall DEM categorization (Type) is based on the DEM ratio, whose validity as a clinical tool has been questioned [35,43,44,45] as well as its ability to predict reading (e.g., [46], but see [15]). In addition, the correlation between DEM scores and eye movements is a topic of discussion [26,47], with some suggesting these measures are not related [43]. In a recent review, Facchin concludes that the DEM likely represents general oculomotor behavior rather than exclusively measuring saccades [26]. It is also not possible to rule out the possibility that DEM measurements also reflect visual attentional skills in conditions resembling reading and that this is partially the cause for the DEM-to-reading relationship found here. An additional issue is the fact that the children tested were at their initial stages of acquiring reading (of words and numbers) and developing the rapid automatic naming (RAN) skill associated with that. Since they may not have been proficient enough in reading or in RAN, the relationship we find between DEM performance and reading may also reflect their lack of proficiency in RAN in both tasks. As the DEM was designed to be read from left to right, and this is how its norms were obtained with native left-to-right language readers (also consistent with the direction of numerical reading), it is unclear whether it can reliably be used to assess oculomotor function in right-to-left language readers. It has been suggested that native language reading direction (left-to-right or right-to-left) may contribute to visual awareness asymmetry (e.g., [48]), and indeed a recent study with Hebrew (right-to-left) speaking children found a directional right-to-left preference in DEM performance (in line with their native reading direction but opposite to that of the DEM instructions and norms [49]). In our study, however, the DEM results were obtained from left-to-right in line with the DEM manual’s instructions and norms but opposite to the children’s natural reading direction (right-to-left). We assume that this difference in reading direction may have led to an overall reduction in DEM performance across our cohort (relative to right-to-left performance), but that DEM performance is likely to be associated across reading directions (even if better for the native language reading direction) and therefore not so likely to affect our findings. Examining the correlation between right-to-left and left-to-right DEM performance in both right-to-left and left-to-right native readers may shed light on this issue in future investigations. Despite all these limitations related to DEM measuring oculomotor performance and the need to further investigate this relation, there is research that is consistent with our findings of a correlation between DEM scores and reading [36,43,50,51]. Furthermore, even if the genuine prevalence of OMD is found to be smaller than that suggested here by the DEM, even halved, this would suggest that a substantial portion of children entering the educational system may have oculomotor-related difficulties, which may be related to reading acquisition difficulties, as our results suggest. It may therefore be beneficial for children who exhibit reading difficulties to potentially undergo testing for OMD in addition to the current tests.

Previous studies that have investigated the relationship between oculomotor skills and reading abilities examined this at later stages of reading acquisition [15,39,43,52] and often the focus was on groups of children with reading-related difficulties (e.g., Refs. [15,39]). For example, a review by Kulp and Schmidt suggests a correlation between oculomotor dysfunction and reading disability across different age groups in an English reading population, and these are based on different oculomotor control assessments than those employed in our study [53]. Raghuram et al. found in a group of developmental dyslexic English readers aged 8–11 years (middle-end of elementary school) that their oculomotor skills as assessed by the DEM were significantly lower than those of typically developing controls [15]. Children struggling with reading may also have longer fixations, a higher number of fixations and saccades, and shorter forward saccades, which is indicative of longer processing times [54,55,56,57]. In our study we examined the relationship between oculomotor function and reading in a normative class at the initial stages of reading acquisition (1st grade, 6–7 years of age) in Hebrew readers (read from right to left) when two different substantiated oculomotor tests (DEM [27,34,35,36,58] and NSUCO [58,59,60,61,62]) were employed. While our results of a relationship between DEM oculomotor measures and reading at the early reading acquisition stage are consistent with these earlier studies and are found in right-to-left readers, suggesting that oculomotor function may be related to reading across languages (regardless of orthographic depth or reading direction), future replications are needed given our small cohort size. In contrast to previous studies in which reading and NSUCO scores were shown to be significantly correlated, our NSUCO findings were weaker than the DEM measures were (see Results and Figure 1, Figure 2 and Figure 5), and this may be due to our small cohort size relative to earlier studies with larger cohorts [59,61]. Furthermore, previous studies investigating the performance of NSUCO and reading ability were performed with English-reading children, whereas our study used Hebrew reading that uses a different (right-to-left) alphabet. Additionally, during the initial reading acquisition of Hebrew, diacritical markers are used to help novice readers pronounce the words correctly, which is very different from English reading acquisition that does not use diacritical markers and instead depends on contextual cues for reading accuracy and correct pronunciation. We hypothesize that the visual demands for the initial stages of Hebrew reading may therefore have different oculomotor demands than those demanded during English reading acquisition. Therefore, the NSUCO test, which has been shown to be an effective nonverbal assessment tool for evaluating gross ocular motor function, may not be sensitive enough to predict reading ability in young Hebrew readers.

In the literature, the relationship between dVA and reading is still unclear. In many countries, including European countries [17] and the USA, visual evaluation in schools often includes only distance visual acuity (dVA), which is effective in detecting amblyopia. A leading cause of reduced dVA is short-sightedness (myopia), and its prevalence in the age range of our cohort is lower than that of far-sightedness (hyperopia) [63,64]. Bruce et al. [65] report that dVA is associated with developing literacy skills at the age of 4–5. In their study they used a subtest of the Woodcock Reading Mastery Tests-Revised (WRMTS-R) that demands letter recognition and not whole word or sentence reading, perhaps due to the young children’s pre-reading age. Such visually local letter recognition may rely on similar functions as VA testing, which may be one of the factors contributing to the correlation they found. A recent review by Hopkins et al. on dVA and reading ability concludes that the impact of dVA on reading ability is still unclear [66], and there are other studies reporting that dVA is not associated with reading ability. For example, one longitudinal study assessing 1143 nine- and ten-year-olds found that there was no correlation between dVA and academic school performance [67]. However, they did not directly assess the relationship between dVA and reading. Additionally, as the age of the children in their study was 9 and 10, it is difficult to draw any inferences from these findings about reading acquisition that typically occurs at an earlier age. Another study also found that dVA was not predictive of reading skills [68]. In our study we also found that dVA was not positively associated with reading acquisition, and our results may be explained by the fact that the reading examination was performed at close viewing distance using large font sizes, while dVA was performed from a far viewing distance. While dVA is important for multiple school-related purposes (seeing the board, reading the teacher’s lips, social communication), the jury is still out about the contribution of distance VA to reading (see also Ref. [69]). In light of our results, we suggest that children who are found to be struggling with reading should be referred to a comprehensive vision evaluation that includes oculomotor assessments. Detection of OMD-related difficulties in early readers could allow consultation with specialists to consider dedicated visual support for improving scholastic performance [12,70,71,72,73,74,75] across school-aged children.

While our results suggest a potential link between oculomotor function and reading ability at early reading stages, there are several limitations that have to be taken into account. First, as already mentioned, the small sample size our results are based on limits the generalization of the results, and future studies with larger cohorts are required to substantiate the findings reported here. Second, there are various limitations associated with the DEM (these are detailed above), and therefore future studies incorporating eye movement measurements are needed to substantiate that DEM measures reflect oculomotor control. Third, we did not separately assess the relative impact of phonological processing on reading, and therefore we cannot determine the extent to which our results may also reflect phonological deficits. To address that, future research should incorporate comprehensive phonological assessments alongside oculomotor evaluations to clarify the respective or potentially combined roles these mechanisms play in early stages of reading acquisition.

## 5. Conclusions

OMD prevalence in school-aged good readers has been suggested to be approximately 15–24% [5,6,15] compared to much higher estimates in children diagnosed with a reading disability [5,7]. Here, retrospectively assessing optometric evaluation results of a normative first grade class, we found that ~29% of the children were categorized by the DEM as having OMD-related difficulties. While future assessments of the relationship between OMD-related measures and reading performance are necessary with larger cohort sizes and direct oculomotor evaluations (e.g., via eye-tracking devices) to corroborate our findings, our study suggests that reading difficulties from near distances (approx. up to 40 cm) among early Hebrew readers may be associated with oculomotor difficulties (i.e., OMD). While there are suggestions that DEM performance reflects visual processing speed and cognitive factors rather than oculomotor performance [45], our results suggest that DEM performance and especially DEM speed (irrespective of the process it actually reflects) is likely associated with reading difficulties in early readers. We propose that future research, including longitudinal studies, should investigate the relationship between oculomotor function and reading abilities during the early stages of reading acquisition. Such studies would help to further clarify the specific and potentially causal contribution of oculomotor function to reading development. Ultimately, this research may pave the way for more precise and individually tailored interventions for learning-related difficulties.

## Figures and Tables

**Table 1:** Descriptive statistics of the group. Value ranges (minimum, maximum, mean, and standard deviation in columns) are detailed for each measurement (in rows). dVA Snellen score of 1 is equivalent to 6/6 (20/20), which is considered to be normative visual acuity. DEM standardized scores have a mean of 100 and an SD of 15, a higher score represents better performance. Individual data are available at https://osf.io/shw2v/ (accessed on 30 May 2025).

Sex	10 girls, 17 boys			
Education	1st grade			
	Min.	Max.	Mean	SD
dVA (Snellen)	0.2	1	0.9	0.26
DEM standardized vertical speed	16	131	85.9	25.28
DEM standardized horizontal speed	−285	126	57.4	87.02
DEM standardized accuracy	59	117	93.4	13.31
DEM standardized ratio	−187	127	68.1	76.84
Reading speed (time in sec)	450	58	180.6	86.91
Reading accuracy (accurate words)	12	75	64.9	13.00

**Table 2:** Linear regression analyses results. Top—models explaining reading accuracy, bottom—models explaining reading speed. Each row specifies one predictor (bottom row is the overall model estimation summary), and each column details the outcome of one linear model with model results included only in cells that were part of that model. In each cell, the model estimate for that variable ± the standard error and, in parentheses, its significance. For DEM, standardized scores were used. Significant values (apart from the intercept) are indicated in bold, asterisks indicate predictor significance (* for *p* < *0*.05, ** for *p* < 0.01, *** for *p* < 0.001).

READING ACCURACY				
	Model 1	Model 2	Model 3	Model 4
**INTERCEPT**	75.43 ± 9.05 (1.52 × 10^−8^ ***)	21.9304 ± 17.3171 (0.2175)	58.74729 ± 3.02448 (3.47 × 10^−16^ ***)	48.62904 ± 22.55831 (0.0423 *)
**DVA**	−12.60 ± 10.11 (0.224)	-	-	−7.13391 ± 9.32139 (0.4522)
**DEM HORIZONTAL ACCURACY**	-	**0.4609 ± 0.1852** (0.0202 *)	-	0.19512 ± 0.24644 (0.4370)
**DEM RATIO**	-	-	**0.08743 ± 0.02936** (0.00654 **)	0.06011 ± 0.04151 (0.1618)
**OVERALL MODEL ESTIMATION**	*F*(1,24) = 1.556, *p* = 0.2243, Adjusted *R*^2^ = 0.02174	***F*(1,24) = 6.194** ***p* = 0.02015** Adjusted *R*^2^ = 0.172	***F*(1,24) = 8.867** ***p* = 0.006543** Adjusted *R*^2^ = 0.2394	***F*(3,22) = 3.245** ***p* = 0.0414** Adjusted *R*^2^ = 0.2122
**READING SPEED**				
	Model 1	Model 2	Model 3	Model 4
**INTERCEPT**	91.0 ± 59.21 (0.137)	212.2101 ± 17.9623 (1.73 × 10^−11^ ***)	304.3051 ± 57.7368 (2.1 × 10^−5^ ***)	232.7528 ± 58.8213 (0.00067 ***)
**DVA**	107.05 ± 66.12 (0.119)	-	-	**117.2505 ± 53.8036** (0.04030 *)
**DEM HORIZONTAL SPEED**	-	−0.5290 ± 0.1724 (0.00526 **)	-	**−1.5051 ± 0.5509** (0.01216 *)
**DEM VERTICAL SPEED**	-	-	**−1.4333 ± 0.6532** (0.0381 *)	**−0.4125 ± 0.1545** (0.01400 *)
**OVERALL MODEL ESTIMATION**	*F*(1,24) = 2.621, *p* = 0.1185, Adjusted *R*^2^ = 0.06089	***F*(1,24) = 9.419,** ***p* = 0.005264**, Adjusted *R*^2^ = 0.2519	***F*(1,24) = 4.815,** ***p* = 0.03813**, Adjusted *R*^2^ = 0.1324	***F*(3,22) = 7.424,** ***p* = 0.001301**, Adjusted *R*^2^ = 0.4353

## Data Availability

The dataset related to this study can be found in the OSF repository at https://osf.io/shw2v/ (accessed on 30 May 2025).
